# Influence on the behavior of lung cancer H1299 cells by silencing SLC35F2 expression

**DOI:** 10.1186/1475-2867-13-73

**Published:** 2013-07-23

**Authors:** Xiao Li, Jilun Li, Guanchao Jiang, Liang Bu, Fan Yang, Jun Liu, Jun Wang

**Affiliations:** 1 Department of Thoracic Surgery, Peking University People’s Hospital, No 11, Xizhimen South, 100044 Beijing, China

**Keywords:** Lung carcinoma, SLC35F2, RNA interference, Lnvasion, Migration

## Abstract

**Background:**

To investigate the effects of RNA interference-mediated downregulation of Human Solute Carrier Family 35 member F2 (SLC35F2) expression on the biological behavior of lung cancer H1299 cells.

**Methods:**

The lentiviral vector of small interfering RNA targeting SLC35F2 was introduced into H1299 cells by liposome-mediated transfection. Expression of the SLC35F2 protein was measured by western blot. The proliferation of H1299 cells was determined by Cell Counting Kit-8 assay. The migration of H1299 cells was measured by Transwell migration assay. Cell cycle analysis used fluorescence-activated cell sorting.

**Results:**

SLC35F2 expression was markedly downregulated in H1299 cell clone (transfected with the lentiviral vector harboring small interfering RNA targeting SLC35F2). Proliferation decreased significantly compared with that of non-transfected H1299 cells. Transwell migration assay showed that fewer cells moved through the artificial basement membrane compared with untransfected H1299 cells (38.3 ± 5.7 vs. 113.5 ± 8.5, P < 0.05). The cell cycle of H1299 cells was changed, the percentage of H1299 cells in S and G2/M phases being significantly decreased compared with untransfected H1299 cells (S phase: 15.3% ± 3.0% vs. 27.0% ± 5.4%, P > 0.05; G2/M phase; 3.0% ± 1.1% vs. 10.5% ± 1.7%, P < 0.05), whereas the percentage of H1299 cells in G0/G1 phase increased markedly (81.7% ± 4.0% vs. 62.5% ± 1.9%, P < 0.05).

**Conclusion:**

RNA interference-mediated downregulation of SLC35F2 expression by lentiviral vector can attenuate the proliferation, migration and invasion of H1299 cells.

## Background

RNA interference (RNAi) is an evolutionarily conserved process whereby double-stranded RNA (dsRNA) induces a sequence-specific degradation of homologous mRNA, leading to post-transcriptional gene silencing. This involves cleavage of dsRNA into short (21–23 nt) small interfering RNAs (siRNAs) with characteristic 2 nt 30-overhanging ends, which is mediated by Dicer.

*Stankovic et al*. [1] initially reported on Human Solute carrier family 35 member F2 (SLC35F2) in ataxia telangiectasia. The SLC35F2 gene is located in the long arm of human 11 chromosome. RAB39, a member of the cancer gene Ras family, is close to SLC35F2 in the same chromosomal position (11q22). It participates in the construction of human blood–brain barrier and has higher expression in adult salivary glands [2,3]. Knowledge about the functions of the SLC35F2 gene is currently lacking. *Shen et al*. [4] found that the SLC35F2 gene might be related to the incidence of lung cancer by differential screening. The Department of Thoracic Surgery in Peking University People's Hospital reported that the expression of the SLC35F2 protein is obviously higher in non-small-cell lung carcinoma (NSCLC) tissue than in normal tissues near the tumor, indicating that SLC35F2 might be a potential oncogene [5,6]. To develop further studies on the biological functions of SLC35F2, we constructed SLC35F2 recombinant RNAi lenti-virus vectors, and got transfected H1299 lung cancer cells in which the expression of SLC35F2 was stably inhibited by RNAi [7]. Here we are to investigate the effect of the SLC35F2 gene on the biological behavior of human lung cancer cell line H1299.

## Results

### Construction and validation of lentiviral vector, establishment of SLC35F2 silencing H1299 cell line

**Figure 1:**
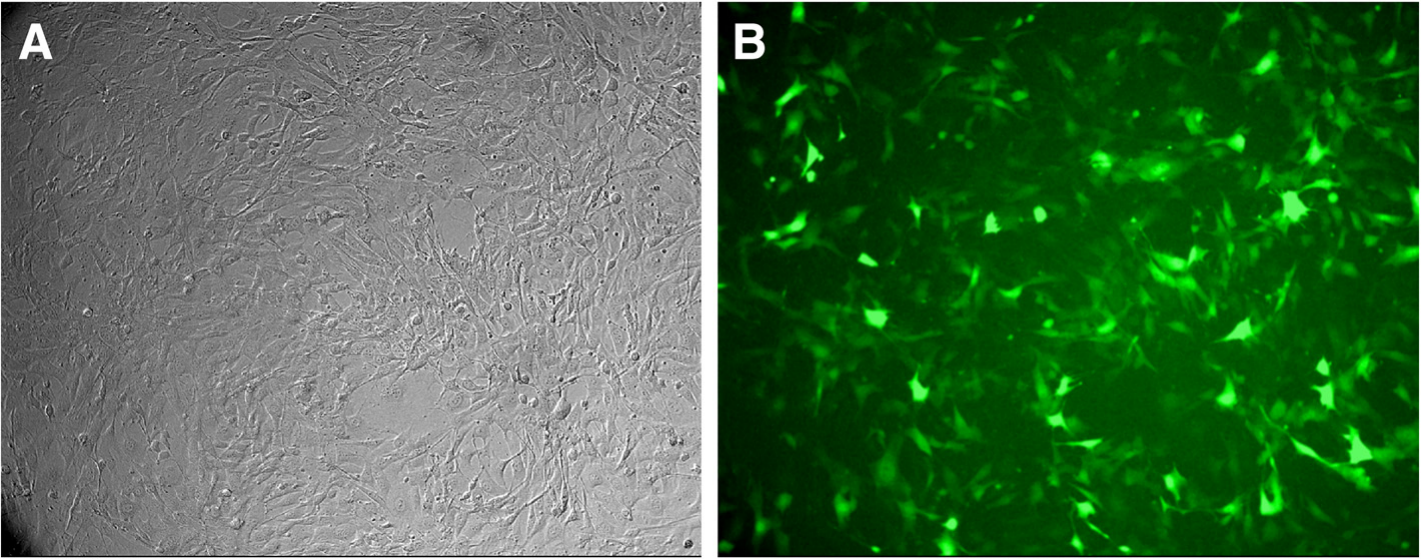
**Expression of green fluorescence protein in H1299 cells after stable transfected under inverted fluorescence microscope(X100). A**: common microscope **B**: fluorescence microscope. We could see the transfected H1299 cell lines with green light under an inverted fluorescence microscope 14 days after the transfection, confirming the transfection is successful and stable.

The lentiviral vector of siRNA targeted against SLC35F2 (SLC-siRNA) was constructed and transfected H1299 cells successfully. We got the H1299 cell lines which were transfected by lentiviral vector harboring specific siRNA (SLC-si) and lentiviral vector harboring control siRNA (SLC-nc) [7]. We could see both of the transfected H1299 cell lines with green light under an inverted fluorescence microscope, confirming the transfection of lentiviral vector to cell genome and its stable expression (Figure 1). Compared with control group, the SLC35F2 expression was decreased significantly both in RNA and protein level, as described in our study before [7].

#### Effect of SLC35F2 on the proliferation capacity of H1299 lung cancer cells

**Figure 2:**
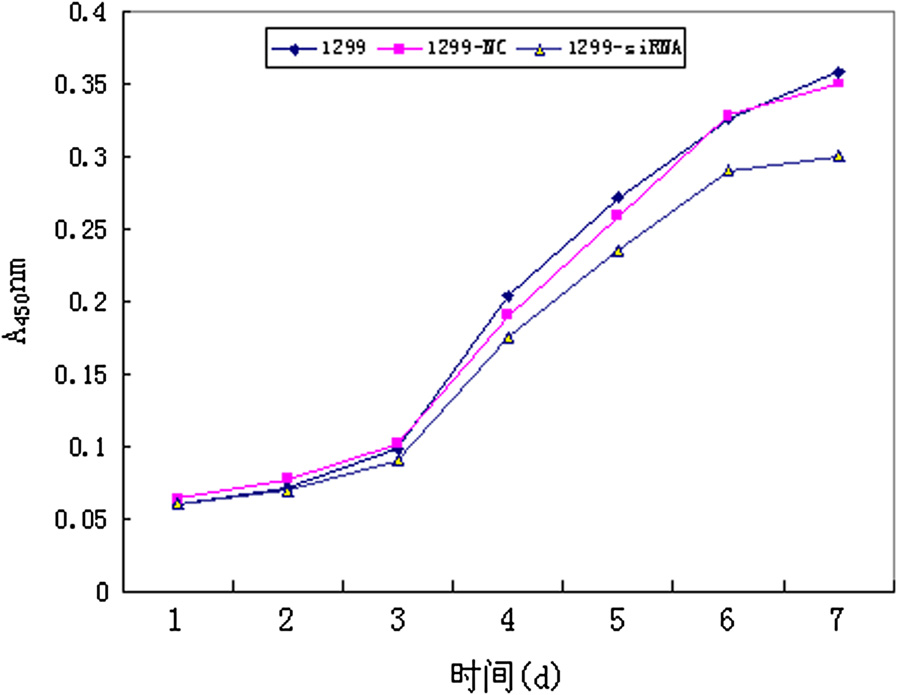
**Growing curves of the different groups. **After 7 days’ culture, the in vitro growth rate of the SLC35F2 suppression group (1299-siRNA) was lower than that of the non-transfected group (1299-NC) (P < 0.05). But no significant difference was found between the negative control group and the non-transfection group (1299).

The effect of SLC35F2 silencing on the proliferation of H1299 lung cancer cells was determined by CCK-8 assay. As shown in Figure 2, after 7 days’ culture, the in vitro growth rate of the SLC35F2 suppression group was lower than that of the non-transfected group (P < 0.05). But no significant difference was found between the negative control group and the non-transfection group (Figure 2). Therefore, in vitro cell growth ability was correlated with SLC35F2 expression.

#### Effect of SLC35F2 on the migration capacity of H1299 cells

**Figure 3:**
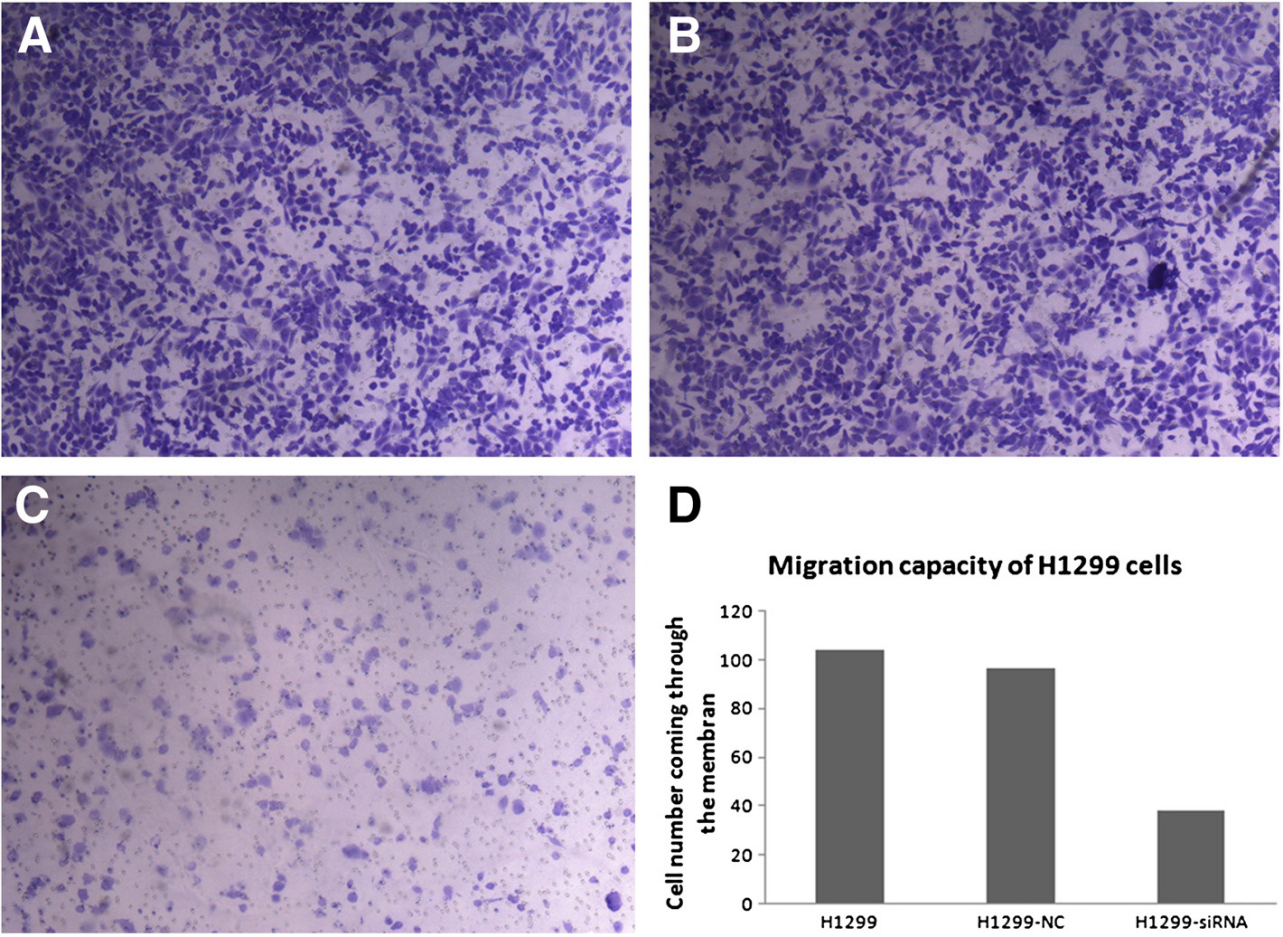
**Migration capacity of different groups of H1299 cells. A**: non-transfected group; **B**: negative control group; **C**: transfected group(X100). **D** Histogram of migration capacity of different groups of H1299 cells. The number of the cells coming through the membrane in group **C **is much less than that of the group **A **and **B**. No significant difference was seen between group **A **and **B**.

After the SLC35F2 gene had been inhibited, the migration capacity of H1299 declined, with the cell population coming through the artificial membrane being less than that of the non-transfected group (38.2 ± 3.7 vs. 103.8 ± 8.5, P < 0.05). No statistical difference in trans-membrane cells was found between the negative control group and the non-transfected group (96.8 ± 9.4 vs. 103.8 ± 8.5, P > 0.05, Figure 3).

#### Effect of SLC35F2 on the invasion capacity of H1299 cells

The tumor cell invasion through the ECM is an important step in tumor metastasis. ECMatrix serves as a reconstituted basement membrane matrix of proteins. The number of cells migrating to ECMatrix was counted. Compared with non-transfected cells, SLC35F2 silencing cells decreased invasiveness significantly (16.5 ± 1.8 vs. 64.5 ± 4.8, P < 0.05). No statistical difference was found between the negative control group and the non-transfected group (58.4 ± 5.2 vs. 64.5 ± 4.8, P > 0.05).

#### Effect of SLC35F2 on the cell cycle of H1299 cells

**Figure 4:**
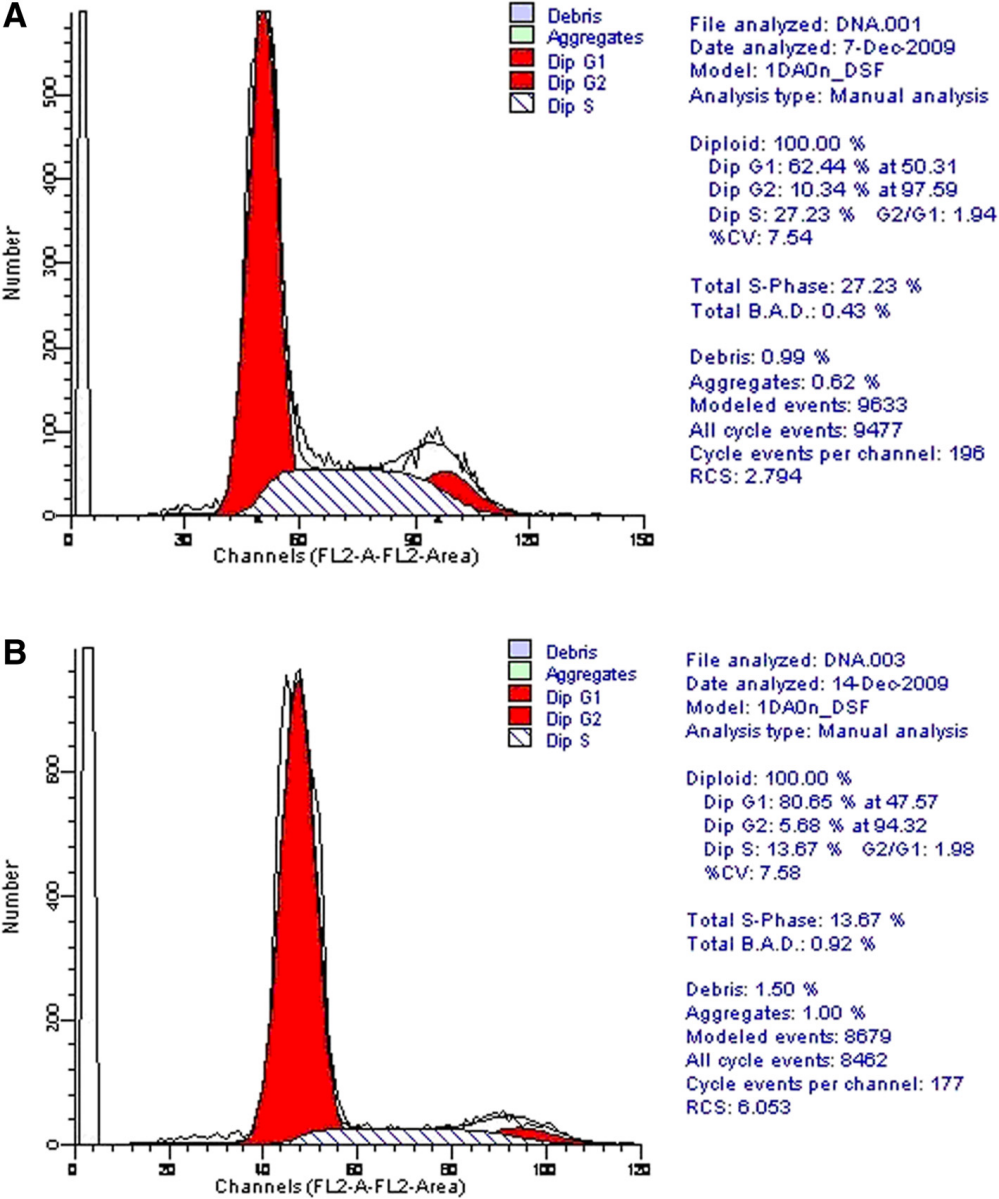
**Effect of SLC35F2 on the cell cycle of H1299 cells. A**: non-transfection group; **B**: transfection group. Compared with untransfected cells, silencing of SLC35F2 caused an accumulation of cells in the G0/G1 phase, while reducing the number of cells in S and G2/M phases. (Here we just show one of the results, all of the experiments measuring cell cycle were performed three times to ensure reproducibility of results, and we got the mean as the final result).

Compared with untransfected cells, silencing of SLC35F2 caused an accumulation of cells in the G0/G1 phase (81.6% ± 4.0% vs. 62.5% ± 1.9%, P < 0.05), while reducing the number of cells in S and G2/M phases(S Stage: 15.3% ± 3.0% vs. 27.0% ± 5.4%, P > 0.05; G2/M Stage: 3.0% ± 1.1% vs. 10.5% ± 1.7%, P < 0.05, Figure 4).

## Discussion

The SLC35 gene, which encodes nucleotide sugar transporter protein, is a hydrophobin consisting of 320 to 400 amino acid residues. It has 6 members: SLC35A-F. SLC35 A-E proteins are located in the endoplasmic reticulum (ER) and Golgi body, with about 10 transmembrane helices. They can transport nucleotide sugar into the Golgi body and/or ER. As sugar donors, they are used to synthesize glycoside-protein, glycolipid, and polyose by glycosyl transferase [8]. As reported, this type of nucleotide sugar transporter is related to neoplasm metastasis, cellular immunity, organ development, and morphological characteristics [9]. According to the study before, SLC35A1 transports CMP-Sia, SLC35A2 transports UDP-Gal, and SLC35A3 transports UDP-GlcNAc [8,10]. SLC35C1 can encode a GDP-Fuc transporter protein [8], SLC35D1 transports UDP-GlcA/UDP-GalNac [11], and SLC35B3 encoded 3′-phosphoadenosine 5′-sulfate transporter protein which is related to the synthesis of sulfated proteoglycan and glycoproteins [12]. The SLC35F family has 5 members (F1-F5), but their functions remain unclear. Matsuyama et al. [13] reported that there was some difference of SLC35F2 expression between 5-fluorouracil-sensitive and resistant tumors.

Studies on the functions of the SLC35F2 gene are currently lacking. The SLC35F2 gene is located in the long arm of human 11 chromosome. RAB39, a member of the cancer gene Ras family, is close to SLC35F2 in the same chromosomal position (11q22). Our previous study showed that the SLC35F2 gene expressed differently in human lung squamous carcinoma and normal tissue nearby. SLC35F2 gene expression is higher in NSCLC tissue than in normal lung tissues near the tumor. So we conjecture SLC35F2 may participate in the occurrence and development of NSCLC, and thus may be a potential oncogene [5,6].

Here we used RNAi to silence SLC35F2 in H1299 cell line, and studied the effect of the SLC35F2 gene on the biological behavior of lung cancer cell line. Proliferation rate was slowed down after silencing SLC35F2, and H1299 cells were blocked in G0/G1 phase, with the number in S phase being reduced significantly. The reason might be that with the reverse transport of nucleotide sugar inside the cells being affected, the synthesis of materials required for proliferation became restricted. The migration and invasion capacities of the cells were reduced after the SLC35F2 gene was inhibited, which suggests that SLC35F2 plays an important role in the migration and invasion of lung cancer cells to peripheral tissues.

For the first time, we have identified the influence of SLC35F2 on the biological behavior of H1299 lung cancer cell line in this study. The results are consistent with our previous studies, and showed SLC35F2 might be a potential oncogene in lung cancer. These results suggest that SLC35F2 has an important role in the occurrence, development, and migration of lung cancer. SLC35F2 might be a potential target for lung cancer therapy in the future.

## Conclusions

RNA interference-mediated downregulation of SLC35F2 expression by lentiviral vector can attenuate the proliferation, migration and invasion capacities of H1299 cells, which suggests that SLC35F2 may be a potential oncogene of lung cancer.

## Materials and methods

### Cell line and cell culture

Human lung squamous cell carcinoma H1299 cell was purchased from Cell Bank of Chinese Academy of Sciences (Shanghai, China), and was cultured in DMEM supplemented 5% fetal bovine serum (Hyclone), and 1% penicillin/streptomycin (Life Technologies).

### Construction of SLC35F2 RNAi vector and transfection

Oligonucleotide sequence of SLC35F2 specific siRNA is pSC-1: CTCTTTCTGTTTGGCTATA. SLC35F2 RNAi lentiviral vector construction, virus infection, cloning and screening, real-time polymerase chain reaction (PCR) and western blot were carried out as described previously [7]. The forward primer 5'- TAACCAGTGTCCAGCTTTTGGA -3', and the reverse primer 5'- CCATGGTTCCTACACCCAACA -3' are used for amplifying SLC35F2.

### Cell growth assay

Dispense 200 μl of H1299 cells suspension (2,000 cells/well) in a 96-well plate. Incubate the plate for 1–7 d at 37°C in an atmosphere of 5% CO2 in air. Add 20 μl of CCK-8 solution to each well of the plate and incubate the plate for additional 4 h at the same condition. Then measure the absorbance at 450 nm using a microplate reader. The absorbance value indicating proliferative capacity. An average value from 3 wells was obtained for each group of H1299 cells to get the growth curve.

### Migration and invasion assays

H1299 cells of nontransfected group, SLC35F2 siRNA lentiviral vector transfected group, and siRNA-C (control) transfected group were detached from culture plates in the absence of trypsin using Hank’s buffered saline solution (HBSS)/5 mM EDTA/25 mM Hepes pH 7.2 (Mediatech). Cells then were washed twice in DMEM and resuspended at a density of 2.5 × 10^5^/ml in DMEM; 200 μl of the cell suspension was added to the upper chamber of an 8 μm pore size Transwell insert (Costar) in triplicate. DMEM culture solution (500 μl) containing 10% fetal bovine serum was added to the lower chamber of each well and incubated for 12 h at 37°C. Cells were fixed with 0.025% glutaraldehyde (Sigma Aldrich) in PBS, stained in 0.1% crystal violet, and nonmigratory cells on the upper surface of the membrane were removed. Membranes were mounted on a microscope slide, and migrated cells were counted in five random high-power fields.

Invasion assays were carried out in a similar manner to migration assays. Transwell inserts with 8-μm pores (Costar) were coated with 200 μl Matrigel (Becton Dickinson), which was diluted 1:6 in ice-cold DMEM, and allowed to gel at 37°C. Sub-confluent cell cultures were detached as described above, resuspended in DMEM, and 1 × 10^5^ cells were seeded in the upper chamber. Culture plates were incubated for 48 h at 37°C, and the cells fixed, stained, and counted as described above.

### Cell cycle assays

H1299 cells were seeded at 2 × 10^5^ cells/well in 6-well plates. To analyze the cell cycle distribution, the cells were collected after 24 h incubation and washed with PBS. The cells were fixed in 70% ethanol and stored overnight at 4°C. For analysis, the cells were transferred into PBS and incubated with Ribonuclease A (50 μg/ml) at room temperature for 5 min. Following incubation, the cells were treated with 10 μg/ml PI and incubated at 37°C for 10 min. Finally, the cells were analyzed using FACS. 1 × 10^4^ cells per experimental condition were analyzed for fluorescence on a Becton-Dickinson FACScan using Cell Quest software. All of the experiments measuring cell cycle were performed three times to ensure reproducibility of results, and we got the mean as the final result.

### Statistical analysis

The software of SPSS version 13.0 for Windows (SPSS Inc, Chicago, IL, USA) was used for statistical analysis. Continuous variables were expressed as X ± s. Statistical analysis was performed with t-test. Differences were considered statistically significant when P was less than 0.05.

## Competing interests

The authors declare that they have no competing interests.

## Authors’ contributions

XL and JL carried out all the molecular genetic studies. GJ and LB participated in the design of the study. FY performed the statistical analysis. JL participated in drafting the manuscript. And JW participated in the study design and coordination. All authors read and approved the final manuscript.
